# A Letter to the Secretary at War on the Sickness and Mortality of the West Indies, &c. &c.

**Published:** 1839

**Authors:** 


### A Letter to the Secretary at War on the Sickness and Mortality of the West Indies, &c. &c. By Sir *A. Halliday*, M.D.

This is chiefly a review of Capt. Tulloch's celebrated statistical report, which we have noticed in a preceding number, and to the merits of which Sir A. bears ample testimony. But introductory to his subject, our author takes occasion to deplore the strange fact, that no medical officer should have been deemed worthy or capable of performing the duty which was thus delegated to a captain in the army destitute of professional knowledge!!

This remarkable and astounding transaction has "tended much to lower the department, already sufficiently low in the estimation of the public, and must prove most disheartening to every member of that department, to find a military subaltern earning promotion by the performance of a task that most especially belonged to the medical officer."

The picture which Sir A. draws of the utter non-attention of military towards medical officers, on all points of hygiene, is absolutely distressing, and we could not have believed in the reality of the portrait, had it not been delineated by an Inspector of Hospitals, who must have the best means of judging. There is one consolation that Capt. Tulloch is acknowledged to have performed the task he undertook with great ability; but still a physician qualified in other respects, might have brought to the investigation some qualifications which Captain Tulloch could not be supposed to possess. At the same time it ought to be observed that Mr. H. Marshall, whom we believe to be a medical man, was associated with Captain Tulloch—a circumstance which would appear to have been overlooked by Sir A. in his complaints on the subject in question.

Our author adverts to certain sources of unhealthiness amongst our troops in the West, and which were pointed out by the medical officers on the spot, but contemptuously despised—because they were pointed out by the Doctor!! One of those was the filthy materials of the beddings,—materials which might have been rendered clean and wholesome, as pointed out by Mr. Gunning. Another cause of sickness—or at least of its prolongation—is the employing of raw young medical officers in the West Indies, who are put in charge of troops without experience.

"Every inducement therefore ought to be held out, not only to stimulate young men of superior abilities to volunteer for this service, but to encourage them to remain in the West Indies as long as their health and constitutions can withstand the climate. At present there is nothing to be gained by any exertion, no stimulus whatever to acquire distinction. However diligent and observing a man may be, or however much he may sacrifice his health or his ease to cure the epidemic and endemial diseases of the colony in which he may be stationed, the prospect of any reward, or even of any praise, is now almost hopeless; nay, I have actually known zeal procure censure." 8.

This is a sorry state of things—and one which will be apt to work its own cure anon!

The third source of sickness is the want of chaplains for the moral instruction of our soldiers, who, instead of attending divine service on Sundays, are strolling about the rum-shops getting drunk.

A fourth cause of sickness and mortality is the paucity of troops :—the consequence of which is, that the men are over-worked and over-watched—so that when sickness or punishments occur, the good men have hardly a whole night in bed ! There are other and minor causes of illness, but we must pass them over.

Our readers are aware that Capt. Tulloch made the average mortality of our troops in the West Indies to be one in eleven. Our author makes it only one in fifteen—the cases of fever being three-eighths of the whole—and the deaths from this disease being one half of the whole. This brings us to the most important topic in the pamphlet—the remedy for fever—"DR. WARBURG'S FEVER DROPS."

"I am most desirous of calling your Lordship's serious attention to these facts, because, if a remedy can be found which will arrest the progress of so general and so fatal a disease, it becomes, in my humble opinion, a matter of national importance, to make it generally known. And that such a remedy has been discovered, I do most firmly and conscientiously believe—one that is of far more value than any preparation of the *quinquinas*, or what is usually called the Spanish or Jesuits' bark, and one whose powers have now been sufficiently proved to warrant its being ordered by authority in the treatment of fever throughout the whole range of our West India and other Colonies.

It was my good fortune to serve for about twelve months in the Colony of British Guiana, where, as Captain Tulloch's returns show, fever is far more prevalent than in any other British Colony ; and where, during what was called the sickly season, from June till November, our hospitals were crowded with every form of the disease. I found that the *sulphate of quinine*, which in Europe and in some of the other colonies had been most efficacious in checking the fever, failed entirely in producing any remission of the symptoms in most of our cases, and when pushed to any extent led to sufferings of an anomalous and distressing kind, that aggravated rather than lessened the violence of the febrile action. In almost every variety of the colonial epidemic, the irritability of the stomach is such as to preclude the exhibition of the bark in substance, or even in the form of a decoction or infusion. Under these circumstances, I was induced to have recourse to a remedy which had already become known in the Colony, and was much talked of as 'DR. WARBURG'S FEVER DROPS.'

I got introduced to the Doctor, who had been resident in Demerara for several years, as a civil practitioner; and soon discovered that he was a physician of very superior talents and a man of keen observation ; a botanist of some distinction and well acquainted with modern chemistry ; and, above all, that he was an enthusiast in science, and often spent months with the Indians in the woods in search of knowledge. The fever drops about which I was anxious, he assured me were prepared from plants, the virtues of which he had ascertained during his sojournings with the native tribes ; and he laid before me evidence which proved that the reports I had heard were not exaggerations. I therefore did not hesitate to direct my able assistant, Dr. Gibson, to give the medicine a fair trial in the regimental hospital of the 25th regiment. It was administered in about fifty of our worst cases, and under my own eye with the most perfect success. Dr. Warburg offered to supply us gratuitously with any quantity we might require, but I felt that as I was not allowed to purchase it for the use of the troops, I had no right to encroach upon the liberality of any individual for the benefit of the public, and was therefore compelled to decline his offer. I did not fail, however, to record what I had witnessed in my official Medical Reports, and I accepted of a supply, which was sent to the Director-General, with a request that trial might be made of its effects in the fevers of Gibraltar and the Mediterranean. I have not heard what the result has been, and possibly it may never have been tried.

There is generally and, I would say, very properly, a great disinclination, on the part of the regular profession, to prescribe remedies of which we know not the composition; but when experience has fully established the character of any drug, or combination of drugs, and it is fairly ascertained that the exhibition of any remedy is uniformly followed by the same effects, in the treatment of the same class of diseases, we are perfectly warranted in adopting it in our *Materia Medica*. And if it shall be found to surpass all other remedies in the cure of that disease, we would, I conceive, become most criminal if we did not employ it, though the preparation may be kept secret. In this case, however, Dr. Warburg is too liberal and enlightened a physician to have any desire for secrecy. He has been at great trouble and great expense in bringing to perfection these invaluable drops, and in distributing them to all quarters of the globe, that they might be tried, and their success or failure in all cases of fever fairly and candidly reported. And, as it now appears, that whenever they have been tried they have succeeded, he thinks he is entitled to some remuneration for the time he has consumed, and the money he has expended in procuring so important a benefit for the whole human race; and that too before he puts others upon an equality with himself in regard to its preparation. I confess, my Lord, there is justice in this: and when your Lordship again refers to the appalling number of cases, 717 in every thousand, of the mean strength of our whole troops! and of the mortality that ensues, I trust you will not feel that I have dwelt too long upon a matter which promises to be of such vital importance in the future treatment of our gallant soldiers, who are subjected to the fearful ravages of West India fever." 23.

Numerous other testimonies from various tropical practitioners have been brought forward by Dr. Warburg, all confirmatory of Sir Andrew's statements. The medicine is now procurable in abundance in London, and it is our intention to try it in some of the various forms of intermittent complaints, short of agues, which are not rare in this country. The intermittent and remittent neuralgia, from the lowest to the highest grade, will be fit subjects for the trial of this remarkable medicine. No one will accuse us of favouring secret nostrums; but if the inventors of remedies will not publish their composition, this is no reason why we should not test their efficacy, when they come from practical and scientific men. Would any man in his senses, or who has any regard for the benefit of his patients, refuse to administer James's powder, Battley's sedative liquor, or Ruspini's styptic, because the inventors refused to make known the composition of these remedies? Dr. Warburg has supplied gratuitously immense quantities of the febrifuge to the Profession, and continues to do so, in order that his statements respecting its properties may be fairly put to the test. This is, perhaps, as much as could be expected under existing circumstances.

Sir A. alludes to the crowded state in which troops are conveyed from one settlement to another—"Men, women, and children all huddled together upon the deck of the army vessel, or of some less comfortable schooner—often without standing-room, and in which condition they are kept for one, two, or three days, exposed to chills, heats, and other vicissitudes." On the benefit or injury of changing the residence of troops in the West Indies, our author offers the following opinion, grounded on facts.

"I investigated the Returns for a great number of years,—followed the movements, and noted the casualties of several regiments, during the whole of their stay in the windward and leeward command,—and it appeared even to me rather surprising, how uniformly all the returns I examined supported each other, and how satisfactorily they proved these great leading facts, namely, *that the more frequently a regiment has been moved from one island to another, or from one station to another*, in the same island or colony, during its residence within the command, *the fewer men it has lost; and that the most stationary corps have always lost the greatest number of men.*" 25.

He thinks that no regiment ought to be kept more than two years in any one island or colony.

"The moral effect of such a certainty (change of air) would have a powerful influence upon the health of both; their sufferings would be endured with patience and resignation, while their spirits would be sustained with hope, and even their pleasures would be enjoyed with a contentment and moderation that would greatly check their running into excess." 29.

The great prevalence of, and mortality from thoracic inflammation, he attributes to the circumstance of the barracks being generally perched on hills, while the towns are at the bases of these eminences.

"Most of the diseases of the lungs, under whatever category they may be placed in Captain Tulloch's tables, arise from inflammatory action induced by the soldiers having to mount a steep ascent to get at their barracks, and where, on their arrival, overcome with heat and exhaustion, they expose their half-covered bodies to the chilling draughts of air that sweep through the galleries where they throw themselves down and fall sleep." 30.

Upon the insalubrious climate of Guiana—"*the wet grave of Europeans*" —Sir A. makes some pertinent observations, and detects, as he avers, some serious errors in Captain Tulloch's calculations and returns, which the parties themselves must settle. We must conclude this short notice of Sir A.'s pamphlet, with the following graphic sketch of Barbadoes.

"Of Barbados, I can speak with more certainty, having well examined it in all its bearings. This island, as is well known, is a mass of solid granite, cased with coral rock, and which evidently has been raised from the bosom of the deep, not by one effort of nature, but by a succession of efforts. The Polypes that formed the pinnacle of Mount Hillaby while it was yet under the wave, may have lived some thousand years before the generations that built the next terrace; and conjecture is lost in imagining the ages that may have elapsed since even the last raised portions of this island assumed their present level. The comminuted portions of the calcareous earth are in general too scanty to admit of a luxuriant vegetation; hence, most of the hills are naked and barren; but opposed to what may be called the current of the ocean, or the force of the waves, as impelled by the constant action of the trade winds, the solid nucleus of Barbados, with its adventitious covering, have assumed the form of a crescent with the points stretching to the north-east and south-east, and forming a deep bay. In this bay the muddy waters, checked in their course, were allowed to deposit the earthy particles they had collected in their progress; and from these particles a considerable extent of solid land has now been formed, of a nature entirely distinct from the great mass of the island—a pure alluvial clay soil, mixed with the thousand and one substances that are usually found in all such recent formations. The surface of this soil is covered with every variety of vegetation and forest trees. It has of course been broken up by the action of the rains, and is now formed into mimic mountains and sequestered valleys, abounding in mineral springs and asphaltic exudations; and, at one spot, affording the singular phenomena of a burning well.

If more attention was paid to the spiritual welfare of the troops, and greater care shown for their temporal comforts, St. Ann's would be as healthy a station as any in Europe. True, it is, there are times and seasons when sickness prevails to a great extent even here, but these are now of rare occurrence. There ought, and eventually *must* be, two regular chaplains commissioned for this garrison, a Protestant and a Roman Catholic: yes, my Lord, a Roman Catholic, for there are seldom fewer than three or four hundred soldiers professing that religion quartered here, and for many years there has not been a clergyman of that persuasion resident on the island." 40.

Our chief object in noticing Sir A.'s Letter was to introduce his testimony respecting Dr. Warburg's febrifuge, and we invite the attention of our brethren in this country to this remarkable preparation. As yet we have only tried it in one case of irregular intermittent, or rather remittent fever, occurring in Belvidere Road on the banks of the Thames. It baffled every remedy which we employed, and the new febrifuge amongst the rest. The patient, however, after the second dose, fell into a perspiration, and without sleeping, expressed himself as having passed the most delightful night he ever experienced. The fever returned the next day, as usual, and as there were some symptoms indicative of congestion in some of the large internal organs, we did not venture on a repetition of the medicine, but trusted to salines and small doses of calomel and James's powder. The febrifuge is the most intense bitter we have ever tasted; and we have little doubt that it is prepared from some of the quinquinas.

